# Acute pulmonary vein isolation lesions consist of interstitial oedema and tissue necrosis: possible mechanism of pulmonary vein reconnection

**DOI:** 10.1186/1532-429X-13-S1-M8

**Published:** 2011-02-02

**Authors:** Aruna Arujuna, Dennis Caulfield, Rashid Karim, Benjamin Knowles, Aldo Rinaldi, Michael Cooklin, Mark ONeill, Kawal Rhode, Jaswinder Gill, Reza Razavi

**Affiliations:** 1 King's College London, London, UK

## Background

Wide area circumferential ablation (WACA) is used to achieve pulmonary vein isolation (PVI) for treatment of AF. AF recurrences associated with pulmonary vein reconnection are common.

## Purpose

We assessed the hypothesis that acute PVI results from a combination of irreversible (necrosis) and reversible (interstitial oedema) tissue damage at the left atrial (LA)-pulmonary vein (PV) junction as evaluated by cardiac magnetic resonance (CMR) imaging.

## Methods

**Figure 1:**
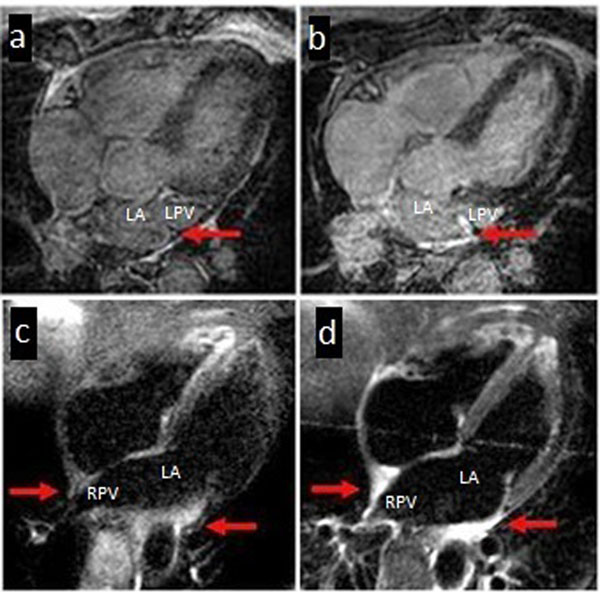
Demonstrating images pre- and post-ablation for late enhancement - figure 1a, 1b and T2-W oedema imaging pre- and post-procedure - figure 1c, 1d.

15 patients with paroxysmal atrial fibrillation (PAF) underwent CMR scanning pre and immediately post WACA. 12 patients (4 male; mean age 56±11 years ) had good quality images for delayed enhancement DE (necrosis) [Figure 1a,1b] and high T2-weighted signal (oedema) [Figure 1c,1d]. Images were analysed to quantify the circumferential extent of lesion formation with both imaging sequences. Clinical follow-up results at 6 months were then correlated with the MR findings.

## Results

Twenty four pairs of pulmonary veins were electrically isolated during the ablation and subsequently divided into no recurrences and recurrences of AF; quantifying the percentage(%) circumferential lesion of DE, high T2-weighted signal and DE + T2. Patients free from AF at follow-up (n=7; 14pairs of PV) had mean ± SD of DE and high T2-weighted signal of 73.2±25.4% and 19.3±21.9% respectively. Patients with recurrences (n=5; 10pairs of PV) had mean ± SD of DE and high T2-weighted signal of 46.5±25.9% and 47.5±28.3%. Between the two groups, the statistical difference in the T2-weighted means (p=0.01) was twice that of the DE means (p=0.02). No statistical difference was seen in the DE+T2 means. On visual assessment areas of high T2 signal (oedema) not only overlapped with areas of DE but also filled in gaps between areas of DE, producing in combination almost circumferential lesions in all PVs.

## Conclusion

CMR scans performed post ablation confirms DE (necrosis) and high T2 signal (oedema) forming a near complete ring around the pulmonary veins contributing to electrical isolation. In patients with recurrent atrial fibrillation, more of this ring was composed of high T2-weighted signal than DE. This may provide evidence for immediate post procedural oedema without necrosis being the mechanism of pulmonary vein reconnection and recurrence of AF post catheter ablation during follow-up.

